# Cuff-Method Thigh Arterial Occlusion Counteracts Cerebral Hypoperfusion Against the Push–Pull Effect in Humans

**DOI:** 10.3389/fphys.2021.672351

**Published:** 2021-06-09

**Authors:** Changyang Xing, Yuan Gao, Xinpei Wang, Wenjuan Xing, Yunnan Liu, Yujia Lei, Xing Zhang, Shu Zhang, Lijun Yuan, Feng Gao

**Affiliations:** ^1^School of Aerospace Medicine, Fourth Military Medical University, Xi’an, China; ^2^Department of Ultrasound Diagnostics, Tangdu Hospital, Fourth Military Medical University, Xi’an, China

**Keywords:** cerebral blood flow, baroreflex, push–pull effect, aviation, hemodynamic

## Abstract

Exposure to acute transition from negative (−Gz) to positive (+ Gz) gravity significantly impairs cerebral perfusion in pilots of high-performance aircraft during push—pull maneuver. This push—pull effect may raise the risk for loss of vision or consciousness. The aim of the present study was to explore effective countermeasures against cerebral hypoperfusion induced by the push—pull effect. Twenty healthy young volunteers (male, 21 ± 1 year old) were tested during the simulated push–pull maneuver by tilting. A thigh cuff (TC) pressure of 200 mmHg was applied before and during simulated push—pull maneuver (−0.87 to + 1.00 Gz). Beat-to-beat cerebral and systemic hemodynamics were measured continuously. During rapid −Gz to + Gz transition, mean cerebral blood flow velocity (CBFV) was decreased, but to a lesser extent, in the TC bout compared with the control bout (−3.1 ± 4.9 vs. −7.8 ± 4.4 cm/s, *P* < 0.001). Similarly, brain-level mean blood pressure showed smaller reduction in the TC bout than in the control bout (−46 ± 12 vs. −61 ± 13 mmHg, *P* < 0.001). The systolic CBFV was lower but diastolic CBFV was higher in the TC bout. The systemic blood pressure response was blunted in the TC bout, along with similar heart rate increase, smaller decrease, and earlier recovery of total peripheral resistance index than control during the gravitational transition. These data demonstrated that restricting thigh blood flow can effectively mitigate the transient cerebral hypoperfusion induced by rapid shift from −Gz to + Gz, characterized by remarkable improvement of cerebral diastolic flow.

## New and Noteworthy

Exposure to acute transition from negative (−Gz) to positive (+ Gz) gravity induces cerebral hypoperfusion, a major risk factor of acceleration-induced loss of consciousness. In this study, we found that restricting thigh blood flow during the rapid shift from −Gz to + Gz could effectively protect against cerebral hypoperfusion mainly through improving cerebral diastolic blood flow and pressure. Our findings may provide effective protective strategy for cerebral perfusion during acute gravitational stress.

## Introduction

The acute change of gravitational gradient may cause dramatic changes of cerebral blood volume and pressure, leading to syncope or even the well-known gravity-induced loss of consciousness (G-LOC) due to cerebral hypoperfusion (Scott et al., 2007; Sheriff et al., 2007). Push—pull maneuver (PPM), a common flight maneuver for pilots of high-performance aircraft, is characterized by a brief exposure to negative gravity along the long (*z*) axis of the body (−Gz) and the subsequent positive gravity (+ Gz) (Banks et al., 1994, 1995). The preceding −Gz stress caused a greater drop of mean arterial blood pressure (MAP) during subsequent + Gz stress than gravitational acceleration from 1 G to + Gz (Banks et al., 1994; Goodman et al., 2000; Hakeman et al., 2003). This push—pull effect increases the risk of G-LOC of pilots when performing PPM (Michaud and Lyons, 1998; Michaud et al., 1998).

The cerebral autoregulation and arterial baroreflex are the primary regulation mechanisms guarding the stability of cerebral blood flow and blood pressure in the face of acute hemodynamic changes (Xing et al., 2017). We have reported that the cerebral autoregulation that remained intact during −Gz responded rapidly and appropriately in a transition to + Gz (Yang et al., 2015). Goodman et al. found that cardiopulmonary and/or arterial baroreceptor activation during the “push” stage initiated peripheral vasodilation, which retarded vasoconstrictive response at the following “pull” stage (Goodman and LeSage, 2002). By applying lower body negative pressure (LBNP) during the −Gz stress, the peripheral vascular response was improved, which enabled a better blood pressure control during simulated PPM (Xing et al., 2020). The subsequent releasing of LBNP at the transition to + Gz stress directly slowed down the blood shift to lower limbs caused by −Gz, thus avoiding the great reduction of blood pressure. This is indicative of the fact that the interruption of blood shift or redistribution between legs and the upper body during the rapid −Gz to + Gz transition could effectively protect the cerebral perfusion during PPM.

Therefore, the key to prevent cerebral hypoperfusion induced by the push–pull effect is to retard the acute blood shift caused by rapid −Gz to + Gz transition. This could be implemented by blocking thigh blood flow using an inflated cuff at the upper thigh. In this study, we hypothesized that the application of thigh cuff (TC) during simulated PPM could counteract the cardio- and cerebrovascular hemodynamic effects and mitigate the reduction of cerebral perfusion at the rapid gravitational transition.

## Materials and Methods

### Ethical Approval

The study conformed to the latest Declaration of Helsinki and was approved by the Ethics Committee of The Fourth Military Medical University. Written informed consents were given by all the subjects.

### Subjects

Twenty healthy young volunteers (male, age 21 ± 1 year, height 173 ± 3 cm, weight 67 ± 5 kg) were recruited from the undergraduate students from our university, who are non-smokers with no history of fainting and/or cardiac arrhythmia or not taking cardiovascular medication. All participants abstained from caffeinated beverages, alcohol, and vigorous exercise at least 24 h before each trial. They all had experienced the tilting test one or two times in class several weeks before the study.

### Experimental Protocol

Measurements were performed on subjects positioned on a computer-controlled tilt table whose transition speed was set to 45°/s (Yang, 2007; Yang et al., 2007). Abdominal belt and shoulder blocks were used to avoid body movement during rapid position transition. Two pressure cuffs were placed at bilateral upper thighs, respectively. The cuffs were designed for adult thigh blood pressure measurement, with a bladder size of 21 × 38 cm, which is suitable for thigh circumference from 46 to 66 cm. A saddle was supplied to prevent the downward shift of body during head up tilt (HUT) to minimize skeletal muscle pump effects. During the test, subjects were coached to avoid leg tensing that causes muscle contraction when measurements were performed as previously described (Xing et al., 2020).

**FIGURE 1:**
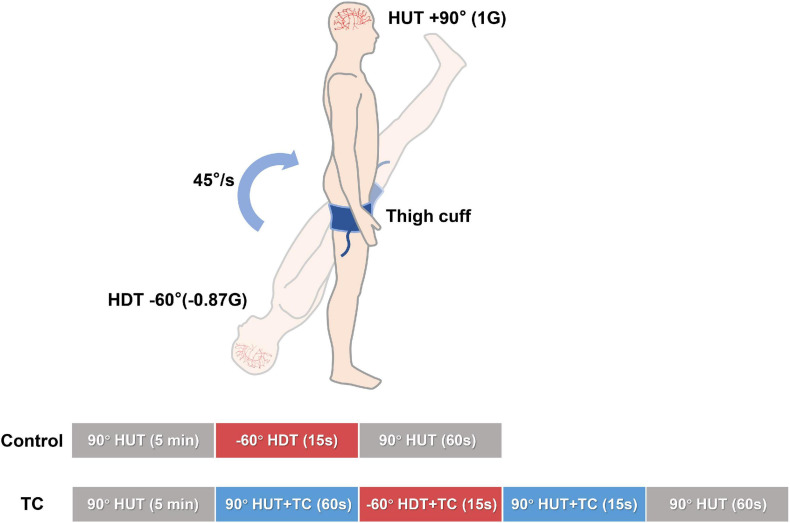
Schema of the study design. Two bouts of HUT—HDT—HUT with or without TC were performed in a randomized design for every subject. The phasic time durations and tilt angles were as marked on the columns. Beat-to-beat cerebral and systemic hemodynamics were continuously recorded. HDT, head down tilt; HUT, head up tilt; TC, thigh cuff.

Before actual testing, familiarization was provided at −60° head down tilt (HDT) for 15 s with a rapid transition to 90° HUT, which minimized psychological responses to the posture change. It appears that the potential influence of this familiarization procedure on the baroreflex-related responses during the following experimental bouts was minor according to a previous report of repeated baroreflex sensitivity measurements during tilt tests (Reynolds et al., 2016). As illustrated in Figure 1, a design of HUT–HDT–HUT was used to simulate PPM. The angle of HUT was set as 90° (1 G). For bout with TC, the cuff pressure was set as 200 mmHg through bladder inflation to interrupt the leg blood flow. TC were applied 60 s before HDT and sustained until the first 15 s of subsequent HUT. In summary, the control bout consisted of 5 min HUT, 15 s HDT, and 1 min HUT; TC bouts consisted of 5 min HUT, 60 s HUT + TC, 15 s HDT + TC, 15 s HUT + TC, and 1 min HUT. A randomized design with alterations of control and TC bouts was used to minimize the potential time effects of repeated gravitational stress.

### Data Acquisition and Process

Heart rate (HR) was recorded using a 3-lead ECG (Dual Bio/Stim, ML408, ADInstruments, Australia). Non-invasive measurement of beat-to-beat arterial blood pressure was performed using finger-cuff plethysmography (Finometer, Finapres Medical Systems, Amsterdam, Netherlands), with height corrected to the heart. Cardiac output (CO) was evaluated by finger arterial pulse wave using Modelflow algorithm that incorporates age, sex, height, and weight to provide stroke volume (SV) estimate (Langewouters et al., 1984). Total peripheral resistance index (TPRi) was calculated as the ratio between beat-by-beat MAP and CO. All signals were outputted at 1,000 Hz from PowerLab (ADInstruments, Australia).

The cerebral blood flow velocity (CBFV) was continuously measured in the middle cerebral artery (MCA) using a transcranial Doppler (EMS-9 PB, Delica, Shenzhen, China) (Xing et al., 2020). A 2-MHz Doppler probe was placed over the temporal window using a headgear (Delica, Shenzhen, China) and fixed at a constant angle and depth where the optimal CBFV signal was obtained as previously described (Xing et al., 2017). Blood pressure at the level of MCA was estimated by subtracting the hydrostatic column between the level of the heart and the transcranial Doppler probe (Yang et al., 2015). Cerebrovascular resistance index (CVRi) was calculated as mean arterial pressure at the level of MCA (MAP_*MCA*_) divided by mean CBFV (CBFVm). Systolic CBFV (CBFVs) and diastolic CBFV (CBFVd) were used to calculate pulsatility index (PI), as (CBFVs−CBFVd)/CBFVm (Xing et al., 2019).

Breath-to-breath CO_2_ was sampled through a nasal cannula and analyzed by an infrared-based carbon dioxide measurement module (CO2100C, BIOPAC Systems, Goleta, CA). Before the trial, participants were instructed to breathe only through their noses with the nasal cannula and avoid any Valsalva-like maneuvers. The breathing of participants was monitored by an experimental assistant. End-tidal CO_2_ (ETCO_2_) values were converted to millimeters of mercury based on atmospheric temperature and pressure.

Baseline HUT data were obtained from the last-minute measurements of the initial 5 min HUT. The 15 s of HDT data were divided to three segments every 5 s. The first 15 s of second HUT data was divided into three segments every 5 s. For the TC bout, the 60-s baseline HUT + TC data were divided into three segments every 20 s. Those specific blocks of time were chosen based on our previous studies about PPM (Yang et al., 2015; Xing et al., 2020) and observation of the original data recordings to better reflect the rapid hemodynamic changes during the protocol as well as to facilitate data analyses.

### Statistical Analysis

*A priori* sample size calculation determined that a minimum of 10 participants would provide sufficient power to detect a difference of 10% ± 10% for change in CBFVm and MAP_*MCA*_ during tilt tests (Sheriff et al., 2007; Yang et al., 2015; Bronzwaer et al., 2017; Xing et al., 2020), with a two-sided α of 0.05. One-way repeated ANOVA was used for the comparisons of the hemodynamic data among different time points during baseline HUT. When significant effect was observed, the *post hoc* paired *t*-test with a Sidak correction was performed between baseline and following TC time points. The paired *t*-test was also performed between control and TC bouts at each single time point. To identify the protection effect of TC, data of the last 5 s of HDT and first 5 s following HUT from control and corresponding TC bouts were analyzed by two-way repeated measures ANOVA with TC and PPM as main factors. When a significant effect was observed, a *post hoc* paired *t*-test with a Sidak correction was performed in the following comparisons: control vs. TC at the last 5 s of HDT; control vs. TC at the first 5 s of following HUT; the last 5 s of HDT vs. first 5 s of following HUT during the control bout; the last 5 s of HDT vs. first 5 s of following HUT during the TC bout. Data were reported as mean ± SD. All statistical analyses were performed with SPSS 20.0 (SPSS, Inc., Chicago, IL). Statistical significance was defined by two-tailed *P* < 0.05.

## Results

None of the subjects experienced loss of peripheral vision, central vision, or consciousness during the experiment based on their self-reports.

### Cerebral Hemodynamics During the Simulated PPM

During baseline HUT, the application of TC increased CBFV in the first 40 s without change of PI (Table 1). Blood pressures at the level of MCA were elevated with TC application (Table 1). CVRi was increased with TC for 40 s (Table 1). No significant changes of ETCO_2_ were found between HUT and HUT + TC (Table 1).

**TABLE 1:** Impact of thigh cuff in the upright posture.

	**HUT**	**HUT + TC**		
	**20 s^#^**	**20 s**	**40 s**	**60 s**
CBFVm (cm/s)	56.9 ± 10.1	64.1 ± 12.5*	59.5 ± 10.7*	57. ± 18.3
CBFVs (cm/s)	86.4 ± 13.1	95.5 ± 15.9*	89.6 ± 13.6*	86.1 ± 11.3
CBFVd (cm/s)	42.1 ± 8.9	48.4 ± 11.3*	44.4 ± 9.4*	42.7 ± 7.2
PI	0.76 ± 0.12	0.74 ± 0.11	0.77 ± 0.09	0.76 ± 0.10
MAP_*MCA*_ (mmHg)	71 ± 7	77 ± 6*	78 ± 7*	77 ± 5*
SBP_*MCA*_ (mmHg)	99 ± 9	110 ± 8*	111 ± 10*	109 ± 7*
DBP_*MCA*_ (mmHg)	56 ± 6	60 ± 5*	61 ± 6*	62 ± 5*
CVRi (mmHg/cm/s)	1.28 ± 0.26	1.24 ± 0.21	1.34 ± 0.22*	1.36 ± 0.21*
ETCO_2_ (mmHg)	36.7 ± 3.6	38.5 ± 3.8	37.9 ± 3.9	37.4 ± 4.3
HR (bpm)	82 ± 13	81 ± 15	79 ± 14	79 ± 14
MAP (mmHg)	93 ± 6	99 ± 6*	100 ± 7*	99 ± 5*
SBP (mmHg)	121 ± 9	133 ± 8*	133 ± 10*	131 ± 7*
DBP (mmHg)	78 ± 6	82 ± 6*	83 ± 6*	84 ± 5*
TPRi (dyn⋅s⋅cm^–5^)	1445 ± 294	1,350 ± 278	1,453 ± 320	1,491 ± 344
SV (ml)	661 ± 3	77 ± 13*	75 ± 15*	74 ± 16*
CO (ml/min)	5,382 ± 1,037	6,218 ± 1,229*	5,847 ± 1,272*	5,704 ± 1,186

One-way repeated ANOVA was used for data comparisons among different time points. When significant effect was observed, the post hoc paired t-test with a Sidak correction was performed between data at baseline and following TC time points. Data were from the baseline HUT and HUT + TC phase of the TC bout. ^#^, the last 20 s of the 5-min baseline HUT measurements. All data are presented as mean ± SD (n = 20). P < 0.05, * vs. baseline HUT. HDT, head down tilt; HUT, head up tilt; CBFVm, mean cerebral blood flow velocity; CBFVs, systolic cerebral blood flow velocity; CBFVd, diastolic cerebral blood flow velocity; PI, Pulsatility index; MAP_*MCA*_, mean arterial pressure at the level of middle cerebral artery; SBP_*MCA*_, systolic blood pressure at the level of middle cerebral artery; DBP_*MCA*_, diastolic blood pressure at the level of middle cerebral artery; CVRi, cerebrovascular resistance index; ETCO_2_, end-tidal CO_2_; HR, heart rate; MAP, mean arterial pressure; SBP, systolic blood pressure; DBP, diastolic blood pressure; TPRi, total peripheral resistance index; SV, stroke volume; CO, cardiac output; TC, thigh cuff.

**FIGURE 2:**
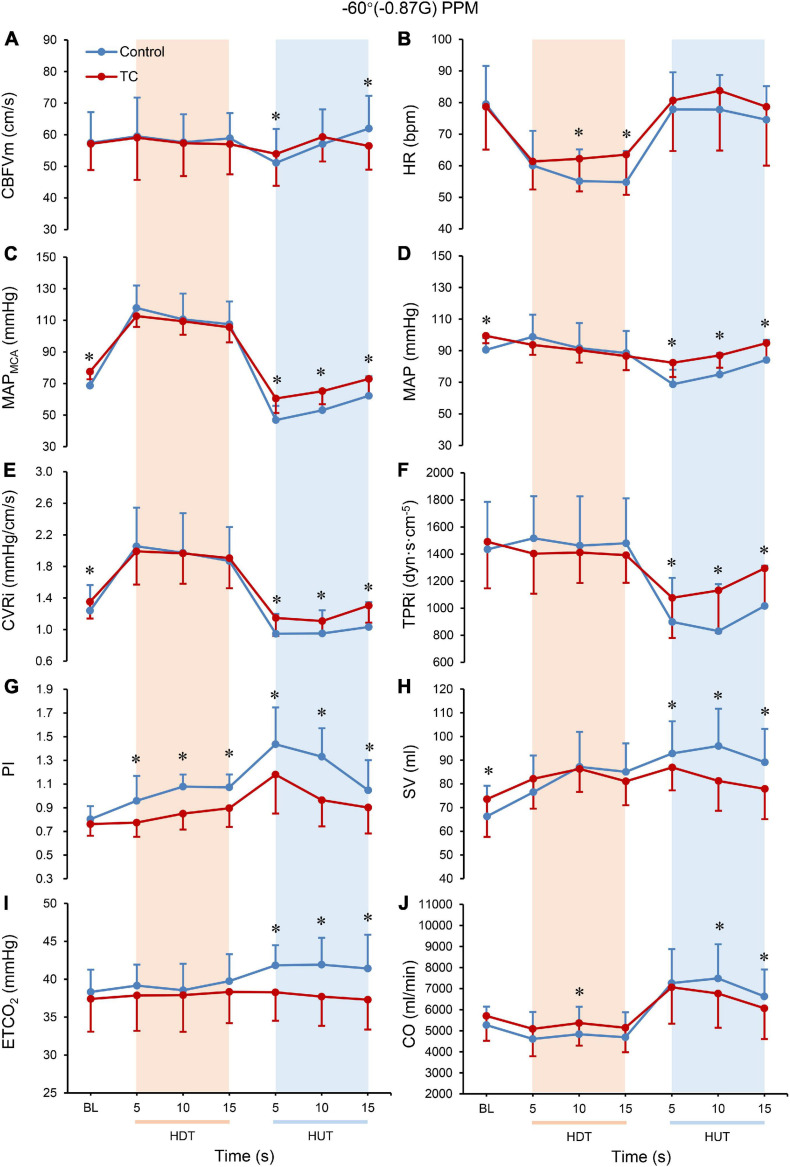
Cerebral and systemic hemodynamic changes during control and TC bouts, including CBFVm **(A)**, HR **(B)**, MAPMCA **(C)**, MAP **(D)**, CVRi **(E)**, TPRi **(F)**, PI **(G)**, SV **(H)**, ETCO2 **(I)**, and CO **(J)**. The blue lines represent the control bout, and the red lines represent the TC bout. The paired *t*-test was performed between control and TC bouts at each single time point. Baseline data were averaged from the last 20 s of the 5-min baseline HUT measurements for control bouts and from the last 20 s of the baseline 60 s HUT + TC measurements for TC bouts. All data are presented as mean ± SD (*n* = 20). ^∗^*P* < 0.05. BL, baseline; CBFVm, mean cerebral blood flow velocity; MAP_*MCA*_, mean arterial pressure at the level of middle cerebral artery; CVRi, cerebrovascular resistance index; PI, pulsatility index; ETCO_2_, end-tidal CO_2_; HR, heart rate; MAP, mean arterial pressure; TPRi, total peripheral resistance index; SV, stroke volume; CO, cardiac output; HDT, head down tilt; HUT, head up tilt; TC, thigh cuff.

During HDT, no significant differences of CBFVm, MAP_*MCA*_, CVRi, or ETCO_2_ were observed between control and TC bouts (Figure 2). PI was lower in the TC bout as compared with the control bout during HDT.

At the rapid −Gz to + Gz transition, the decrease of CBFVm was smaller in the TC bout than the control bout (ΔCBFVm, control vs. TC bouts, −7.8 ± 4.4 vs. −3.1 ± 4.9 cm/s, *P* < 0.001) (Table 2). CBFVs was lower but CBFVd was higher in TC bouts than control bouts during this rapid transition (Table 3). MAP_*MCA*_ dropped significantly in both bouts, while the TC bout showed smaller reduction in MAP_*MCA*_ than the control bout (ΔMAP_*MCA*_, control vs. TC bouts, −61 ± 13 vs. −46 ± 12 mmHg, *P* < 0.001) (Table 2). CVRi demonstrated significantly smaller decrease in the TC bout than in the control bout during HDT-HUT. PI remained lower in the TC bout than in the control bout during the rapid −Gz to + Gz transition (Figure 2), with significant effect of TC × PPM interaction (*P* = 0.021, Table 2). ETCO_2_ was elevated in the control bout, while it remained quite stable in the TC bout.

**TABLE 2:** Cerebral and systemic hemodynamics during the rapid −Gz to + Gz transition.

		**−60°(−0.87G) PPM**		***P*-values**		
		**HDTend**	**HUTbegin**	**TC**	**PPM**	**TC × PPM**
CBFVm (cm/s)	Control	58.9 ± 8.0	51.1 ± 10.7^†^	0.805	< 0.001	< 0.001
	TC	57.0 ± 9.5	53.9 ± 10.1^†^*			
MAP_*MCA*_ (mmHg)	Control	108 ± 14	47 ± 9^†^	0.039	< 0.001	< 0.001
	TC	106 ± 10	60 ± 9*^†^			
CVRi (mmHg/cm/s)	Control	1.87 ± 0.43	0.95 ± 0.25^†^	0.022	< 0.001	0.002
	TC	1.91 ± 0.38	1.15 ± 0.23*^†^			
PI	Control	1.07 ± 0.11	1.44 ± 0.31^†^	< 0.001	< 0.001	0.021
	TC	0.90 ± 0.16*	1.18 ± 0.33*^†^			
ETCO_2_ (mmHg)	Control	39.7 ± 3.5	41.8 ± 2.6^†^	0.002	0.010	0.007
	TC	38.3 ± 4.1*	38.3 ± 3.7*			
HR (bpm)	Control	55 ± 10	78 ± 12^†^	0.009	< 0.001	0.116
	TC	64 ± 13*	81 ± 16^†^			
MAP (mmHg)	Control	89 ± 14	69 ± 9^†^	0.039	< 0.001	< 0.001
	TC	87 ± 9	82 ± 9*^†^			
TPRi (dyn⋅s⋅cm^–5^)	Control	1,480 ± 331	899 ± 325^†^	0.399	< 0.001	0.017
	TC	1,392 ± 204	1,077 ± 296*^†^			
SV (ml)	Control	85 ± 12	93 ± 14^†^	0.102	0.024	0.573
	TC	81 ± 10	87 ± 10*^†^			
CO (ml/min)	Control	4,688 ± 1,193	7,261 ± 1,617^†^	0.590	< 0.001	0.084
	TC	5,141 ± 1,165	7,063 ± 1,726^†^			

Data of the last 5 s of HDT and first 5 s of the following HUT from control and corresponding TC bouts were analyzed by a priori two-way repeated ANOVA with TC and PPM as main factors. When a significant effect was observed, a post hoc paired t-test with a Sidak correction was performed: control vs. TC at HDTend; control vs. TC at HUTbegin; HDTend vs. HUTbegin during the control bout; HDTend vs. HUTbegin during the TC bout. All data are presented as mean ± SD (n = 20). P < 0.05, * vs. Control; ^†^ vs. HDTend. HDTend, the last 5 s of head down tilt; HUTbegin, the first 5 s of head up tilt; PPM, push—pull maneuver; TC, thigh cuff; CBFVm, mean cerebral blood flow velocity; MAP_*MCA*_, mean arterial pressure at the level of middle cerebral artery; CVRi, cerebrovascular resistance index; PI, Pulsatility index; ETCO_2_, end-tidal CO_2_; HR, heart rate; MAP, mean arterial pressure; TPRi, total peripheral resistance index; SV, stroke volume; CO, cardiac output.

**TABLE 3:** Systolic and diastolic hemodynamics during the rapid −Gz to + Gz transition.

		**−60°(−0.87G) PPM**		***P*-values**		
		**HDTend**	**HUTbegin**	**TC**	**PPM**	**TC × PPM**
CBFVs (cm/s)	Control	101.5 ± 16	99.2 ± 16.1	< 0.001	0.910	0.088
	TC	91.0 ± 13.2*	95.2 ± 14.7			
CBFVd (cm/s)	Control	37.6 ± 4.8	27.1 ± 10.3^†^	0.006	< 0.001	0.001
	TC	39.9 ± 8.6	33.2 ± 11.1^†^*			
SBP_*MCA*_ (mmHg)	Control	137 ± 12	83 ± 11^†^	0.199	< 0.001	< 0.001
	TC	134 ± 10	93 ± 10*^†^			
DBP_*MCA*_ (mmHg)	Control	93 ± 17	29 ± 9^†^	0.024	< 0.001	< 0.001
	TC	91 ± 10	44 ± 9*^†^			
SBP (mmHg)	Control	118 ± 11	105 ± 11^†^	0.199	0.021	< 0.001
	TC	115 ± 10	115 ± 9*			
DBP (mmHg)	Control	74 ± 16	50 ± 9^†^	0.024	< 0.001	< 0.001
	TC	72 ± 9	66 ± 9*^†^			

Data of the last 5 s of HDT and first 5 s of the following HUT from control and corresponding TC bouts were analyzed by a priori two-way repeated ANOVA with TC and PPM as main factors. When a significant effect was observed, a post hoc paired t-test with a Sidak correction was performed: control vs. TC at HDTend; control vs. TC at HUTbegin; HDTend vs. HUTbegin during the control bout; HDTend vs. HUTbegin during the TC bout. All data are presented as mean ± SD (n = 20). P < 0.05, * vs. Control; † vs. HDTend. HDTend, the last 5 s of head down tilt; HUTbegin, the first 5 s of head up tilt; PPM, push—pull maneuver; TC, thigh cuff; CBFVs, systolic cerebral blood flow velocity; CBFVd, diastolic cerebral blood flow velocity; SBP_*MCA*_, systolic blood pressure at the level of middle cerebral artery; DBP_*MCA*_, diastolic blood pressure at the level of middle cerebral artery; SBP, systolic blood pressure; DBP, diastolic blood pressure.

During HUT following −60° HDT, CBFVm of the TC bout was higher than the control bout at the first 5 s and back to the baseline level at 15 s, while CBFVm of the control bout continued to increase (Figure 2). MAP_*MCA*_ and CVRi were higher with TC over the 15 s following −60° HDT. PI and ETCO_2_ were lower in the TC bout than in its control.

### Systemic Hemodynamics During the Simulated PPM

During baseline HUT, the systemic blood pressures, SV, and CO were all increased with TC applied, while no significant change of HR or TPRi was observed (Table 1).

During HDT, MAP, TPRi, and SV were not different between control and TC bouts. HR was higher in the TC bout than its control at 10 s and 15 s (Figure 2). CO was slightly elevated at 10 s in the TC bout during −60° HDT.

At the rapid −Gz to + Gz transition, HR was elevated similarly in response to the HDT–HUT transition for both bouts. MAP decreased by 22% in the control bout, but only 6% in the TC bout (Table 2). The TC bout showed much smaller reduction of TPRi than the control bout. SV and CO were raised similarly in both bouts.

During HUT following −60° HDT, HR was similar between the control bout and TC bout. MAP and TPRi were higher with TC over the 15 s following the simulated PPM (Figure 2). SV and CO were decreased in the TC bout compared with the control bout.

## DISCUSSION

The current study was designed to explore an effective strategy to counteract cerebral hypoperfusion induced by abrupt transition from −Gz to + Gz. We found that cuff-method thigh blood flow restriction could effectively mitigate the decrease in cerebral blood flow and arterial blood pressure due to simulated PPM and protect the cerebral perfusion, with predominant preservation of the diastolic cerebral blood flow and pressure. This strategy also improved systemic blood pressure control, characterized by systemic blunted blood pressure response combined with similar HR increase, smaller decrease, and earlier recovery of TPRi in the TC bout than the control bout during the simulated PPM.

The dramatic reduction of MAP_*MCA*_ and cerebral perfusion during transition from “pull” (−Gz) to “push” (+ Gz) constitutes the direct reasons for loss of vision or conscious during PPM (Scott et al., 2007). The observed reduction of cerebral blood flow during the simulated PPM was caused by the dramatic and acute drop of blood pressure. Both the magnitude of blood pressure changes and the time scale in which they occur are too large and too fast to be fully corrected by autoregulation. The cerebral autoregulation itself was not affected in healthy young subjects during the rapid gravitational transition (Yang et al., 2015). Blood flow restriction *via* a TC at the preceding −Gz phase and the subsequent transition to + Gz in the simulated PPM of the current study effectively reduce the drop of MAP_*MCA*_ and cerebral blood flow. This can be explained by the fact that the blood volume redistribution between legs and upper body was prevented by the inflated cuffs during HDT and at the initiation of HUT. In control bouts, the decreased blood volume in the legs caused by HDT would be expected to persist during the initial period of subsequent HUT (owing to the venous valves) (Sheriff et al., 2007). Therefore, the pressure gradient was increased between the upper body and legs during the early HUT, resulting in the greater increase of venous return in legs and fall in MAP_*MCA*_ (Sheriff et al., 2007). TC interrupted the blood shift between legs and upper body, counteracting the changes of leg blood volume at both HDT and HUT. Therefore, the pressure gradient between heart and leg was reduced, which in turn facilitated the initially increased SV distributing to the brain. In contrast with the decreased CBFVm of the LBNP countermeasure during HDT, the TC strategy in the present study showed similar CBFVm with the control. This is probably due to the lower ETCO_2_ and MAP_*MCA*_ in our previous LBNP countermeasure during HDT (Xing et al., 2020), while ETCO_2_ and MAP_*MCA*_ were similar between TC and control in the present study.

**FIGURE 3:**
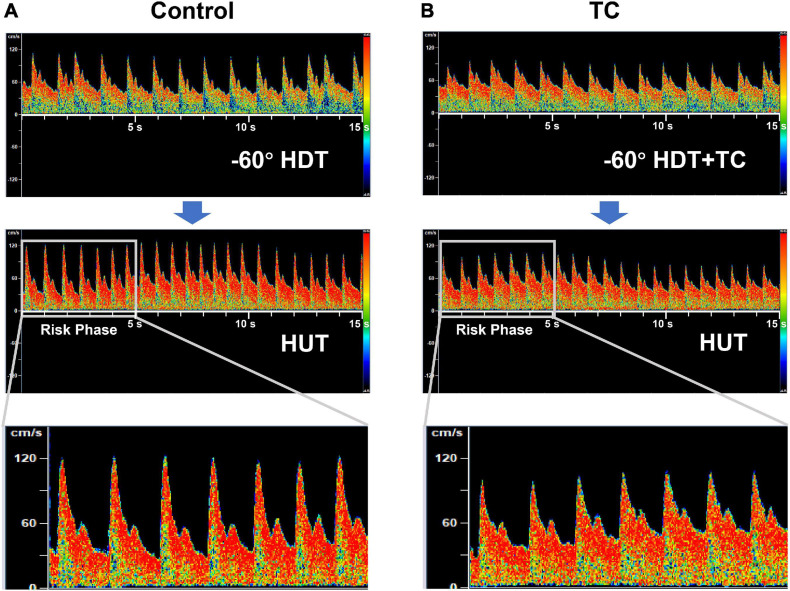
Representative transcranial Doppler records of cerebral blood flow velocity during the rapid HDT-to-HUT transition in control **(A)** and TC **(B)** bouts. The duration of each record in the top and middle panel is 15 s. The first 5-s records of cerebral blood flow velocity during HUT after the rapid transition were zoomed in the bottom panel, as the loss of consciousness in pilots during PPM generally happens just after the rapid transition from −Gz to + Gz (risk phase). HDT, head down tilt; HUT, head up tilt; TC, thigh cuff; PPM, push—pull maneuver.

TC not only improved the CBFVm and MAP_*MCA*_ but also decreased the pulsatility of cerebral blood flow during the simulated PPM compared with the control bout. The significantly higher increase of pulsatility in the control bout was owing to the obvious reduction of diastolic cerebral blood flow (Figure 3), which was also observed in clinical syncope (Jorgensen et al., 1993; Van Lieshout et al., 2003). The reduction of diastolic flow in the control bout might be caused by the markedly larger drop of DBP_*MCA*_ than SBP_*MCA*_ (%ΔDBP_*MCA*_ vs.%ΔSBP_*MCA*_, 69% vs. 39%, Table 3). On the contrary, the cerebral blood flow and pressure at the diastolic phase was well protected by TC (Figure 3 and Table 3). The diastolic phase is much longer than systolic phase during the cardiac cycle. Therefore, even if the systolic flow is maintained, the reduced flow during the longer diastolic phase may be insufficient to maintain the brain oxygen supply, leading to loss of vision or G-LOC during acute gravitational transition. The significant improvement of diastolic cerebral blood flow by TC could be helpful in the maintenance of vision and consciousness during PPM.

The preserved MAP in the TC bout compared with the control bout during the rapid gravitational transitions indicated an improved blood pressure control by TC. It has been reported that peripheral vasodilation due to cardiopulmonary and/or arterial baroreceptor activation during “push” stage retards vasoconstrictor response during the following “pull” stage (Goodman and LeSage, 2002). Using TC during HDT could unload baroreceptors and improve the peripheral vascular response, to achieve a better blood pressure control in the vascular arm (Ogoh et al., 2002; Kaufmann et al., 2020). On the other hand, TC applied at the transition directly impedes blood shift toward legs caused by HUT, thus maintaining the blood pressure at a higher level. Similarly, the increased leg vascular resistance by TC during HUT could directly increase the total peripheral resistance (as shown by TPRi) (Sheriff et al., 2007). Our observation of an earlier recovery of TPRi in the TC bout after transition was in accordance with the more rapid central control of the baroreflex mentioned above. The similar HR response in combination with the blunted SBP response suggested that the cardiac arm of baroreflex might be also improved in the TC bout (Xing et al., 2017; Kaufmann et al., 2020).

Both TC and control bouts showed similar initial SV increase in response to abrupt −Gz to + Gz transition, probably because blood flow to the heart discharged from the apical segments of the lung, and/or left ventricular filling increase due to reduced right atrial pressure *via* ventricular interdependence (Sheriff et al., 2007, 2010; Xing et al., 2013). After 5 s following −Gz to + Gz transition, SV decreased in the TC bout, whereas it continued to increase until 10 s in the control bout. It is suggestive that TC decreased blood reserve in the lungs during HDT. The control and TC bouts shared similar CO changes at the rapid transition from −Gz to + Gz, while the TC bout showed improved TPRi response than the control bout. Therefore, the improved cerebral blood velocity response to leg arterial occlusion is more likely attributed to the increased peripheral vascular resistance.

The anti-gravity suits, or the MAST trousers, all aim at shifting the blood volume from the lower limbs and/or abdomen and pelvis to the upper body to increase the central circulation, which would exacerbate the “push” effect (−Gz) (Fraser et al., 1994; Eiken et al., 2002; Scott et al., 2007; Chatham and Strecker-McGraw, 2020). The anti-gravity straining maneuver, which combines a Valsalva-like strain and a peripheral musculoskeletal isometric strain, could increase both the blood pressure and venous return (Latham et al., 1991). Similar to the anti-G suit, the increased venous return from legs by anti-G straining maneuver also aggravates the −Gz hemodynamics during the “push” stage. Therefore, both the current G-suits and anti-G maneuvers are not suitable for PPM protection. Our thigh arterial occlusion method aims at preventing blood redistribution between legs and the upper body by local cuff compression at the upper thighs, which counteracts not only the “push” effects caused by the cranial shift of leg blood flow but also the “pull” effects due to the caudal shift of central blood, i.e., biphasic counteractive effects. It is easily integrated into the current anti-gravity suits to offset their incapacity of −Gz protection. Furthermore, the TC countermeasure might also be used to counteract the rapid drop of blood pressure and decrease of cerebral blood flow at syncope episode in patients with autonomic failure (Sheldon et al., 2015; Arnold et al., 2017). The TC countermeasure, if developed into some wearable and portable devices, could be used in the daily management for patients with autonomic syncope during acute posture changes (e.g., quickly standing after lying down, sitting, or bending). To minimize the initial psychological stimulus, a tutorial experience before the formal application in patients with autonomic failure was suggested.

There are several limitations that should be mentioned. First, the present study only enrolled male subjects. A previous centrifuge study found Gz tolerances overall to be the same in men and women (Gillingham et al., 1986). However, the reports about gender-based differences in orthostatic tolerance evaluated by LBNP were still controversial. Lawler et al. reported that no influence of the obvious differences in leg mass between genders on the LBNP tolerance was found (Lawler et al., 1998), while Convertino (1998) and Gotshall (2000) found that women have a lower orthostatic tolerance in the LBNP test. It is also well known that gender has a distinct influence on arterial pressure regulation (Hart et al., 2009; Joyner et al., 2016). Thus, it is likely that the protection effects of present thigh arterial occlusion may vary between different genders, which merits further investigation. Second, we did not study the potential effect of age on the results. In our previous study, it was demonstrated that the blood pressure and cerebral blood flow variability in older adults during sit—stand maneuvers are augmented compared with the young and middle-aged (Xing et al., 2017). Therefore, it is likely that the rapid −Gz to + Gz transition during PPM may cause larger hemodynamic changes in the older subjects; whether the TC method in the present study could achieve effective protection on the cerebral perfusion needs further study. Third, we did not monitor the detailed state of vision by objective methods. Although none of the subjects experienced loss of peripheral or central vision during the experiment, the acute changes of acceleration forces during flight maneuver may also cause impairments in color and night vision (Tipton et al., 1984; Balldin et al., 2003). Finally, as the hemodynamic changes were very fast during the transitions in the simulated PPM, the time duration was too short to achieve satisfied direct imaging assessment of the cardiac chamber volumes such as echocardiography. More sophisticated echocardiography or other imaging methods in the future will provide better evidences for the mechanisms affecting the stroke volume in the transitions during PPM.

## Conclusion

Blood flow restriction by TC applied immediately before and during transition from −Gz to + Gz could afford effective protection against the reduction of cerebral blood flow and pressure, which was mainly attributable to the diastolic protective effect.

## Data Availability Statement

The original contributions presented in the study are included in the article/supplementary material, further inquiries can be directed to the corresponding author/s.

## Ethics Statement

The studies involving human participants were reviewed and approved by the Ethics Committee of The Fourth Military Medical University. The patients/participants provided their written informed consent to participate in this study.

## Author Contributions

CX, YG, and FG contributed to conception and design of the study. CX, YG, XW, WX, YLiu, and YLei performed the study. CX and YG organized the database. CX performed the statistical analysis. CX wrote the first draft of the manuscript. YG, WX, XZ, SZ, LY, and FG wrote sections of the manuscript. All authors contributed to manuscript revision, read, and approved the submitted version.

## Conflict of Interest

The authors declare that the research was conducted in the absence of any commercial or financial relationships that could be construed as a potential conflict of interest.
